# The Effect of Iloprost and N-Acetylcysteine on Skeletal Muscle Injury in an Acute Aortic Ischemia-Reperfusion Model: An Experimental Study

**DOI:** 10.1155/2015/453748

**Published:** 2015-03-05

**Authors:** Osman Tiryakioglu, Kamuran Erkoc, Bulent Tunerir, Onur Uysal, H. Firat Altin, Tevfik Gunes, Selim Aydin

**Affiliations:** ^1^Department of Cardiovascular Surgery, Bahcesehir University Medical Faculty, Istanbul, Turkey; ^2^Department of Cardiovascular Surgery, Medical Park Bursa Hospital, Bursa, Turkey; ^3^Department of Cardiovascular Surgery, Osmangazi University Medical Faculty, Eskisehir, Turkey; ^4^Vocational School of Health Services, Osmangazi University Medical Faculty, Eskisehir, Turkey; ^5^Department of Cardiovascular Surgery, Mehmet Akif Ersoy GKDCEA Hospital, Istanbul, Turkey; ^6^Department of Cardiovascular Surgery, Pamukkale University Medical Faculty, Denizli, Turkey; ^7^Department of Cardiovascular Surgery, Acibadem Atakent Hospital, Istanbul, Turkey

## Abstract

*Objective*. The objective of this study was to examine the effects of iloprost and N-acetylcysteine (NAC) on ischemia-reperfusion (IR) injuries to the gastrocnemius muscle, following the occlusion-reperfusion period in the abdominal aorta of rats. *Materials and Methods*. Forty male Sprague-Dawley rats were randomly divided into four equal groups. *Group 1: control group. Group 2 (IR)*: aorta was occluded. The clamp was removed after 1 hour of ischemia. Blood samples and muscle tissue specimens were collected following a 2-hour reperfusion period. *Group 3 (IR + iloprost)*: during a 1-hour ischemia period, iloprost infusion was initiated from the jugular catheter. During a 2-hour reperfusion period, the iloprost infusion continued. *Group 4 (IR + NAC)*: similar to the iloprost group. *Findings*. The mean total oxidant status, CK, and LDH levels were highest in Group 2 and lowest in Group 1. The levels of these parameters in Group 3 and Group 4 were lower compared to Group 2 and higher compared to Group 1 (*P* < 0.05). The histopathological examination showed that Group 3 and Group 4, compared to Group 2, had preserved appearance with respect to hemorrhage, necrosis, loss of nuclei, infiltration, and similar parameters. *Conclusion*. Iloprost and NAC are effective against ischemia-reperfusion injury and decrease ischemia-related tissue injury.

## 1. Introduction

Ischemia is the absence or insufficiency of blood supply to tissues due to various reasons. Hypoxic tissue damage occurs as a result of ischemia. Long periods of ischemia lead to a loss of cellular integrity and result in cellular death. Reperfusion, on the other hand, is the return of the blood supply to the tissue. During reperfusion, the release of free oxygen radicals, especially from the polymorphonuclear leukocytes (PMNL) that accumulate in the tissue, accelerates tissue breakdown. This is referred to as reperfusion injury [1]. Different biochemical methods are used to determine tissue injury, and TAS and TOS are among the well-known methods. Additionally, these markers were comprehensively evaluated in the reports of previous experimental peripheral ischemia studies [2].

Local and systemic effects that are caused by the ischemia-reperfusion syndrome are frequently seen in surgery and affect the postoperative morbidity and mortality. It is known that ischemia-reperfusion injury develops in various tissues, including skeletal muscle, heart, lung, central nervous system, kidney, and gastrointestinal system. Acute ischemia-reperfusion injury in the lower extremities appears particularly during temporary cross-clamp application to the abdominal aorta or during unilateral/bilateral acute femoral artery congestion [2–5]. During abdominal aorta surgery, local tissue injury can occur in ischemic extremities and distant organ injury can occur in other regions. Local effects are seen in distal regions of the clamp, veins, and muscle tissue, whereas systemic effects are seen mainly in the brain, heart, lung, kidneys, and other organs. In clinical practice, a clamp is placed on the infrarenal aorta during abdominal aortic aneurysm, Leriche syndrome, and aortoiliac diseases, and the lower extremity stays ischemic until anastomosis is completed. Following anastomosis and the removal of the aortic clamp, reperfusion of ischemic tissues occurs [3–5].

The objective of this study was to investigate the effects of iloprost (a Pgl2 analog) and NAC on ischemia-reperfusion injuries to the gastrocnemius muscle following an occlusion-reperfusion period in infrarenal abdominal aorta of rats.

## 2. Materials and Methods

The study was approved by the Eskisehir Osmangazi University Animal Experiments Local Ethics Committee (26.04.2011, approval number 212/2011). Animals were purchased from the Medical and Surgical Experimental Research Center (MSERC). Forty male Sprague-Dawley rats (mean weight 300–350 g) were divided into four equal groups (*n* = 10). Animals were kept in an environment with a 12:12 h light-dark cycle and constant temperature (20–22C°) and humidity (45–50%). During the experiments, all animals were kept in transparent cages, were fed with standard rat diet, and were given tap water.

All animals were weighed before the procedures and their body weights were recorded. After 8 hours of starvation, all animals received 50 mg/kg of ketamine hydrochloride anesthesia intramuscularly (Ketalar 50 mg/mL flacon, Pfizer). Median laparotomy was performed after achieving the appropriate position and establishing vascular access. Approximately 10 mL of saline solution was injected into the peritoneal space for fluid resuscitation. Infrarenal abdominal aorta was explored by obtuse dissection. Low dose heparin (100 U/kg) (Nevparin 25000 IU 5 mL flacon, Mustafa Nevzat) was administered. An atraumatic microvascular clamp was placed on the infrarenal abdominal aorta (Nova 12 mm Angle). To minimize heat and fluid loss, the abdominal cut was closed with silk sutures. Following 1 hour of ischemia, 2 hours of reperfusion period was performed. During the procedure, 0.9% saline solution (10 mg/kg) was administered intravenously to provide hydration. Aortic ischemia was monitored with the loss of pulsation in the aorta after clamping; reperfusion was monitored by the presence of pulsation in the aorta after clamp removal. In the control group, laparotomy and infrarenal abdominal aorta dissection were performed for the same time period; however, the ischemia-reperfusion period was not performed. All animals were sacrificed after reperfusion. Blood samples were collected from the right ventricle, and tissue specimens were collected from the gastrocnemius muscle. Muscle tissue specimens were stored in 10% formaldehyde solution.

### 2.1. Experimental Groups

#### 2.1.1. Control Group (Group 1) (*n* = 10)

Laparotomy was performed, but the aorta was not occluded. Saline solution was injected into the abdomen, and the abdominal cut was closed with a silk suture. One-hour ischemia and 2 hours of reperfusion period, which was performed in other groups, were completed.

#### 2.1.2. Ischemia-Reperfusion Group (SHAM) (Group 2) (*n* = 10)

The aorta was rotated with an obtuse dissection, and an atraumatic microvascular clamp was placed. The presence of pulsation under clamp was checked. Saline solution was injected into the abdomen, and abdomen was closed with a silk suture. After 1-hour ischemia, silk sutures were removed. The microvascular clamp was removed, and the presence of pulsation in the aorta was checked by palpation. Saline solution was injected into the abdomen. Abdominal layers were closed again. Following 2 hours of reperfusion period, blood and tissue samples were collected, and the animals were sacrificed.

#### 2.1.3. Ischemia-Reperfusion + Iloprost Group (Group 3) (*n* = 10)

During a 1-hour ischemia period, 2 mcg/kg/h iloprost (Bayer Schering Pharma AG, Berlin-Wedding, Germany) infusion was initiated from the jugular catheter at the 50th minute of ischemia [6]. The microvascular clamp was removed after the ischemia period. The presence of a pulse in the aorta was checked. Saline solution was injected into the abdomen. Then, the abdomen was closed again, and iloprost infusion continued throughout the 2 hours of the reperfusion period. At the end of the procedure, blood and tissue samples were collected, and the animals were sacrificed.

#### 2.1.4. Ischemia-Reperfusion + NAC Group (Group 4) (*n* = 10)

Similar to the iloprost group, during the 1-hour ischemia period, 20 mg/kg NAC (Sandoz Ltd, Basel, Switzerland) was administered as a bolus loading dose via the jugular catheter at the 50th minute of ischemia [7]. Infusion was initiated at a dose of 20 mg/kg/h.

Drug dosages were determined according to previous studies [6, 7].

### 2.2. Biochemical Analyses

Blood samples were kept at room temperature for 30 minutes, and plasma samples were separated and were stored at −70C°. Then, plasma samples were allowed to thaw at room temperature. Total antioxidant capacity (TAC), total antioxidant activity (TAA), total antioxidant status (TAS), creatine kinase (CK), and lactate dehydrogenase (LDH) levels were measured. Reactive oxygen species are produced during metabolic and physiological processes and harmful oxidative reactions can occur in organisms that eliminate these molecules through enzymatic and nonenzymatic reactions. Under certain circumstances, the increase in oxidants and the decrease in antioxidants cannot be prevented, and more than 100 disorders, such as oxidative stress, develop.

Antioxidant molecules prevent or eliminate these harmful reactions. It is possible to measure serum or plasma concentrations of different antioxidants individually; however, these measurements require a significant amount of time and labor and involve expensive and complex techniques. Given that the measurement of different antioxidant molecules is not practical and the additive effects of antioxidants, total antioxidant capacity is measured and has been named differently, including total antioxidant capacity (TAC), total antioxidant activity (TAA), and total antioxidant status (TAS). The TAS measurement method is a fully automated method developed by Erel and is capable of measuring the total antioxidant capacity of the body against strong free radicals [2, 8, 9].

### 2.3. Total Antioxidant Status (TAS)

The test is based on the reduction of the color intensity that is provided by the blue-green ABTS^+^ (2.2′-azinobis-3-ethylbenzothiazoline-6-sulfonate) radical, upon the addition of antioxidants to the medium. To produce the ABTS^+^ radical, ABTS is incubated with metmyoglobin (HX-Fe^+^), a peroxidase, and H_2_O_2_. The resulting ferryl myoglobin reacts with ABTS to produce the ABTS^+^ radical. The ABTS^+^ radical is partially stable and has a blue-green color. The color change is inhibited depending on the amount of antioxidants in the sample. This color change is measured in 600 nm wavelength. A vitamin E analog, Trolox, was used as a standard in TAS measurement [2, 8].

### 2.4. Total Oxidant Status (TOS)

The TOS measurement method is a fully automated, colorimetric method developed by Erel. The oxidant molecules within a sample oxidize the ferrous ion-o-dianisidine complex to ferric ion. The presence of glycerol accelerates the rate of this reaction approximately threefold. Ferric ions form a colored complex with xylenol orange in an acidic environment. The color intensity depends on the amount of oxidant molecules within the sample and is measured spectrophotometrically. Hydrogen peroxide is used for calibration, and the results are expressed as *μ*mol H_2_O_2_ equiv./L [2, 9].

### 2.5. Histopathological Examination of Muscle Tissues

The gastrocnemius muscle samples were fixed in 10% neutral formaldehyde solution. Then, the samples were dehydrated in gradually increasing alcohol series, became transparent in xylol, and embedded in paraffin. Five-micrometer-thick sections were put on poly-L-lysine-coated slides and stained with hematoxylin and eosin. Tissue samples were visualized on a photomicroscope (Olympus, with a DP70 digital camera).

Tissue injury was scored semiquantitatively as follows: (0) no injury, (1) mild, (2) moderate, and (3) severe. Injury was evaluated with respect to the following parameters: loss of striation in muscle fibers, faint peripheral nuclei, necrosis, cellular swelling, edema and fibrosis, leukocyte accumulation in capillaries between muscle fibers, congestion, hemorrhage, and PMNL, monocyte, and macrophage infiltration.

### 2.6. Statistical Analysis

Kruskal-Wallis one-way variance analysis was used to evaluate the differences between the groups. Tukey's multiple comparison test was used to evaluate the difference of each group from other groups. SPSS v.15.0 and Sigma Stat v.3.10 software were used for statistical analysis. *P* values <0.05 were considered statistically significant.

## 3. Findings

### 3.1. Biochemical Findings

Serum TAS, TOS, CK, and LDH levels are shown in Table 1.

When the results were evaluated using the Kruskal-Wallis one-way variance analysis, there was a significant difference between all groups (*P* < 0.05) (Table 1).

Tukey's multiple comparison test showed that the mean TAS level was highest in Group 1 and lowest in Group 2. In Group 3 and Group 4, the mean TAS levels were lower compared to Group 1 but higher compared to Group 2 (*P* < 0.05). In addition, there was no significant difference between Group 3 and Group 4 (*P* > 0.05) (Table 2).

The mean TOS, CK, and LDH levels were highest in Group 2 and lowest in Group 1. In Group 3 and Group 4, these levels were lower compared to Group 2 but higher compared to Group 1 (*P* < 0.05). When Group 3 and Group 4 were compared, the mean TOS level was significantly lower in Group 3 compared to Group 4 (*P* < 0.05).

### 3.2. Histopathological Findings

The results of the histological examination in the control group and experimental groups are shown in Table 2.

Tissues were stained with hematoxylin and eosin and visualized on a photomicroscope (Olympus, equipped with a DP70 digital camera).

**Figure 1:**
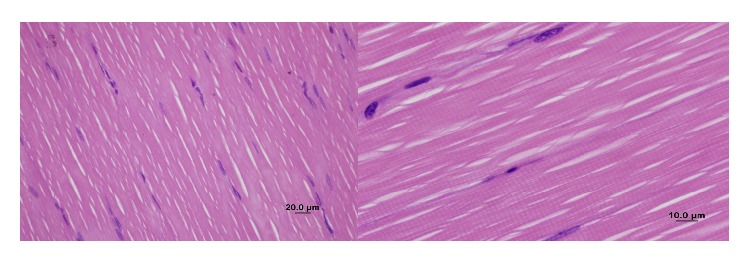
Normal appearance of the gastrocnemius muscle in the control group (H&E staining, bar 20 *μ*m, bar 10 *μ*m).

The control group (Group 1) had normal muscle tissue appearance (Figure 1). Peripheral nuclei in muscle fibers were not faint. Loss of striation in muscle fibers, cellular swelling, necrosis, edema, and interfibrillar fibrosis were not observed. There was no sign of leukocyte accumulation between the interfibrillar capillaries. There were no signs of congestion or hemorrhage. PMNL infiltration and monocyte-macrophage infiltration were not observed.

**Figure 2:**
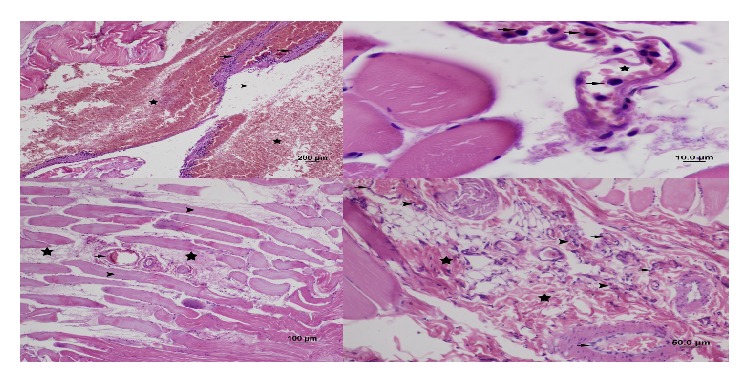
Appearance of the gastrocnemius muscle in the ischemia-reperfusion group: loss of striation in muscle fibers, cellular swelling and necrosis (star), edema between muscle fibers and fibrosis (arrow head) (H&E staining, bar 100 *μ*m), edema between muscle fibers (arrow), leukocyte infiltration and fibrosis, congestion in capillaries (arrow head) (H&E staining, bar 50 *μ*m), cellular swelling and necrosis (star), edema between muscle fibers, leukocyte infiltration and fibrosis (arrow head), congestion in capillaries (H&E staining, bar 20 *μ*m), cellular swelling and necrosis (star), edema between muscle fibers (arrow head), monocyte and macrophage infiltration (arrow), fibrosis, and congestion in capillaries (H&E staining, bar 10 *μ*m).

**Figure 3:**
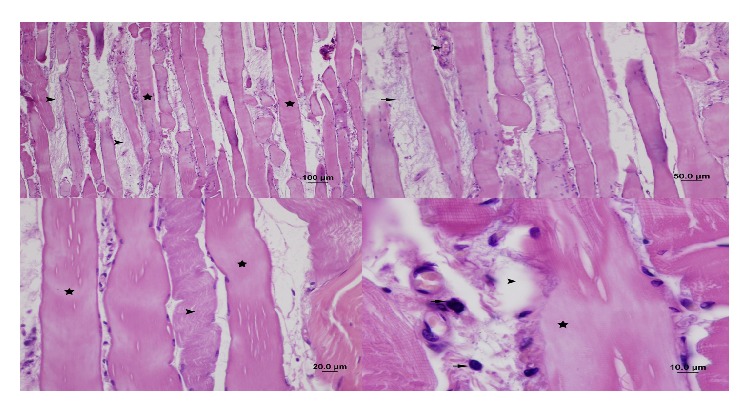
Appearance of the gastrocnemius muscle in the ischemia-reperfusion group: edema between muscle fibers (arrow head), leukocyte infiltration (arrow), congestion in capillaries and hemorrhage (star) (H&E staining, bar 200 *μ*m), leukocyte infiltration between muscle fibers (arrow), congestion in capillaries (star) (H&E staining, bar 10 *μ*m), loss of striation in muscle fibers, cellular swelling and necrosis (arrow head), edema between muscle fibers (star), leukocyte infiltration, fibrosis, congestion in capillaries (arrow) (H&E staining, bar 100 *μ*m), leukocyte infiltration (arrow head), fibrosis (star), and congestion in capillaries (arrow) (H&E staining, bar 50 *μ*m).

In the ischemia-reperfusion group (Group 2), peripheral nuclei were not faint. Loss of striation in muscle fibers and cellular swelling were mild; necrosis, edema, and interfibrillar fibrosis were moderate. Leukocyte accumulation in capillaries between the fibers was moderate. Congestion was moderate, whereas hemorrhage was not observed. PMNL infiltration was severe, and monocyte-macrophage infiltration was moderate (Figures 2 and 3).

**Figure 4:**
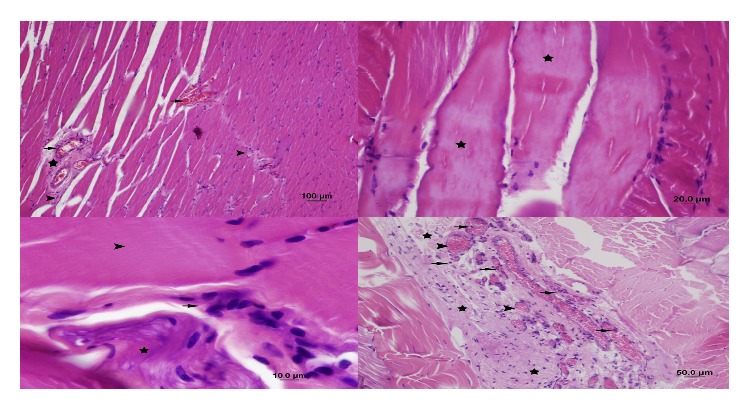
Appearance of gastrocnemius muscle in the iloprost group: congestion in capillaries (arrow) and leukocyte infiltration between muscle fibers (arrow head). Reduction in edema and fibrosis (star) (H&E staining, bar 100 *μ*m), cellular swelling and necrosis (star) (H&E staining, bar 20 *μ*m), cellular swelling, necrosis (arrow head), leukocyte infiltration in muscle fibers (arrow), fibrosis (star) (H&E staining, bar 10 *μ*m), leukocyte infiltration between muscle fibers (arrow) and fibrosis (star), and congestion in capillaries (arrow head) (H&E staining, bar 50 *μ*m).

**Figure 5:**
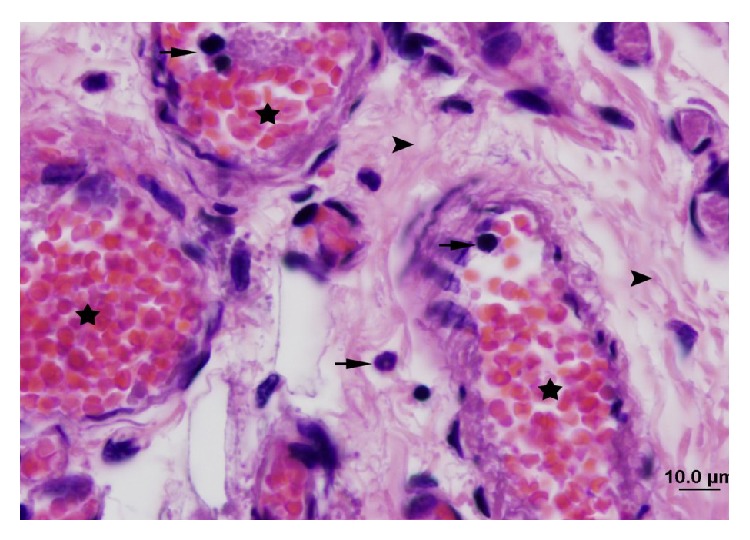
Appearance of gastrocnemius muscle in the iloprost group: leukocyte infiltration between muscle fibers (arrow) and fibrosis (arrow head) and congestion in capillaries (star) (H&E staining, bar 10 *μ*m).

In the iloprost group (Group 3), there was moderate loss of striation in the muscle fibers. Peripheral nuclei were not faint. There was no sign of cellular swelling in fibers, whereas necrosis, edema, and fibrosis were moderate. Interfibrillar leukocyte accumulation was moderate. Congestion was moderate, and there was no sign of hemorrhage. PMNL infiltration and monocyte-macrophage infiltration were mild (Figures 4 and 5).

**Figure 6:**
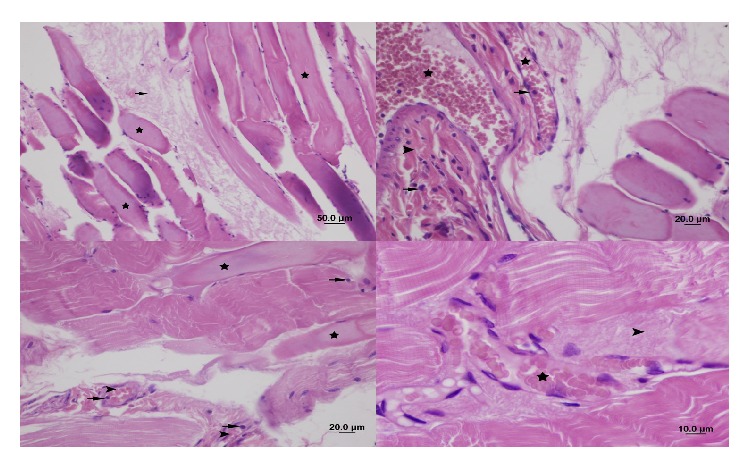
Appearance of the gastrocnemius muscle in the NAC group: loss of striation in muscle fibers, necrosis (star), and fibrosis (arrow) (H&E staining, bar 50 *μ*m). Fibrosis (arrow head), congestion in capillaries (star), and leukocyte infiltration (arrow) (H&E staining, bar 20 *μ*m). Loss of striation in muscle fibers (star), cellular swelling, necrosis, congestion in capillaries (arrow head), and leukocyte infiltration (arrow) (H&E staining, bar 20 *μ*m). Interfibrillar fibrosis (arrow head) and congestion in capillaries (star) (H&E staining, bar 10 *μ*m).

In the NAC group (Group 4), loss of striation in muscle fibers was moderate. Peripheral nuclei were not faint. Cellular swelling and necrosis in fibers were mild; edema and fibrosis were moderate. Leukocyte accumulation in interfibrillar capillaries, congestion, and PMNL and monocyte-macrophage infiltration were mild. There was no sign of hemorrhage (Figure 6).

When we evaluated the results (Table 3), there was a significant difference in the loss of striation in muscle fibers between Group 1 and Group 2 (*P* < 0.05). A significant difference was not observed between the other groups.

There was no significant difference in faint peripheral nuclei between the groups. When we compared the groups for necrosis, cellular swelling, and edema there was a significant difference between only Group 1 and Group 2 (*P* < 0.05).

There was a significant difference in fibrosis development between Group 1 and Group 2 and Group 1 and Group 4 (*P* < 0.05).

There was a significant difference in leukocyte accumulation in capillaries between Group 1 and Group 2 (*P* < 0.05).

There was a significant difference in congestion between Group 1 and Group 2 and between Group 1 and Group 3.

There was no significant difference in hemorrhage between the groups (*P* > 0.05).

There was a significant difference in PMNL and monocyte and macrophage infiltrations between Group 1 and Group 2 and between Group 2 and Group 4 (*P* < 0.05).

## 4. Discussion

Several studies have been carried out on the effect of iloprost on skeletal muscle injury, heart injury, lung injury, and spinal cord injury related to ischemia-reperfusion [6, 10–16]. Saçar et al. and Tekin et al. have shown that iloprost decreases ischemia-reperfusion injury in muscle tissue [10, 13]. On the other hand, Aytacoglu et al., Yakut et al., and Ozcan et al. have shown that iloprost effectively decreases ischemia-reperfusion injury in kidney tissue [14–16]. Myocardial tissue injury and edema after ischemia/reperfusion were investigated in a recent ischemia-reperfusion model by Caliskan et al. They reported the possible protective role of iloprost against ischemia-reperfusion injury in rat myocardium histopathologically [6].

Iloprost is a PgI2 analog, has pharmacological features of its endogenous precursor, and is more stable compared to its endogenous precursor. Iloprost decreases neutrophil adhesion. It prevents the release of free radicals from neutrophils, thus decreasing the endothelial injury caused by neutrophils during reperfusion. Endogenous PgI2 and nitric oxide are autacoids that decrease vascular resistance and increase blood flow. Similar to its endogenous precursor Pgl2, iloprost has strong cytoprotective, antiaggregant, and vasodilator effects. It disrupts the leukocyte-endothelial association by inhibiting thrombocyte aggregation and decreasing the synthesis of adhesion molecules. As a result, it increases microcirculation and removes microvascular dysfunction. In addition, we believe that iloprost effectively decreases muscle injury after ischemia-reperfusion, by increasing tissue perfusion and creating an endothelial-protective effect. Overall, iloprost decreases ischemia-reperfusion-related muscle and distant organ injury, similar to its endogenous precursor [17–19]. Our findings indicate that iloprost causes a significant decrease in ischemia-reperfusion injury in the muscle tissue.

NAC is used as an antioxidant as it increases intracellular glutathione synthesis and also is a direct free oxygen radical scavenger. Previous studies on the protective effects of antioxidants against ischemia-reperfusion injury have also increased our interest in antioxidants. In this study, we preferred to use NAC as an antioxidant, as it is of low cost, easily obtainable, and easy-to-use. In addition, its common clinical use in the treatment of bronchopulmonary diseases and bronchial secretion disorders was an incentive [20–24].

The first clinical observation on NAC was conducted by Sochman and Peregrin in 1992. The authors described the first use of NAC in a patient with acute myocardial infarction, during their effort to protect left ventricle function with pharmacological and mechanical treatment (thrombolysis and percutaneous transluminal coronary angioplasty) and to minimize infarct area [25]. This comprehensive treatment led to the restoration of suppressed left ventricle systolic function. In 1990, Sochman et al. demonstrated the cardioprotective effect of NAC in dogs. The left anterior descending coronary artery was tied for 2 hours before its first diagonal branch. At the end of this period, 100 mg/kg of NAC was administered to the treatment group. In conclusion, the authors showed that NAC decreased the frequency of ventricular arrhythmia after reperfusion and decreased the infarct area [25, 26].

A study in 1996 showed that NAC can be used to minimize ischemia-reperfusion syndrome in patients who undergo abdominal aortic aneurysmectomy. In this study, the patients received 150 mg/kg of NAC 30 minutes before a clamp was placed on the infrarenal aorta. Following clamp removal, a significant increase in plasma reduced glutathione (GSH) concentration was detected in these patients. Moreover, there was no change in plasma lipid peroxide levels [27].

Fischer et al. demonstrated that NAC administration to dogs increases the solubility of myocardial edema after cardioplegic arrest and cardiopulmonary by-pass and protects the systolic functions. Ten minutes prior to the cardiopulmonary by-pass, NAC was administered at a bolus dose of 100 mg/kg, and then NAC infusion was continued at a dose of 20 mg/kg/h until 1 hour after cardiopulmonary by-pass. In conclusion, following cardioplegic arrest and cardiopulmonary by-pass, oxidative stress decreased in dogs that were administered NAC [28].

Creatine kinase (CK), lactate dehydrogenase (LDH), myoglobin, aspartate aminotransferase (AST), brain natriuretic peptide (BNP), atrial natriuretic peptide (ANP), carbonic anhydrase, troponin, and structural muscle proteins are used to determine skeletal muscle injury. Among these proteins, CK has the most frequently used enzyme in the clinic and has the highest sensitivity to muscle injury. In this regard, LDH is the second most common protein. CK is responsible for ATP replenishment in muscle contraction or transport systems. During each contraction cycle, the skeletal muscle uses creatine phosphate to synthesize ATP. Thus, the ATP levels in the muscle remain constant. CK functions like a catalyzer in this reversible reaction. In the present study, the plasma CK levels increased significantly in the acute ischemia-reperfusion group. This finding reflects the ischemia-reperfusion injury in the gastrocnemius muscle following aortic occlusion-reperfusion. Several experimental and clinical studies have shown that plasma CK levels increase following acute ischemia-reperfusion [29–33]. We found that plasma CK levels in IR + iloprost (Group 3) and IR + NAC (Group 4) groups were significantly lower compared to the IR group (Group 2). In a similar study, the effect of iloprost on skeletal muscle injury after acute ischemic-reperfusion was investigated, and the CK levels were significantly lower in the IR + iloprost group compared to the IR group.

Saçar et al. also placed a cross-clamp on the infrarenal abdominal aorta of rats for 3 hours and performed 1 hour of reperfusion [10]. During reperfusion, iloprost infusion was performed through the internal jugular vein, and blood samples were collected after reperfusion. Then, CK and LDH levels were analyzed, and similar to our study, the authors found that the CK and LDH levels were significantly lower in the IR + iloprost group compared to the IR group.

In our study, the significant decrease in plasma CK levels in the IR + iloprost group is consistent with similar studies. Thus, it is possible to say that iloprost decreases skeletal muscle injury after ischemia-reperfusion.

Oxidative stress depends on the disequilibrium between reactive oxygen species and antioxidant defense systems and the decrease in synthesis/activity of antioxidant enzymes [2, 34, 35]. Individual measurement of oxidant molecules is not a practical approach, and it is also known that oxidant molecules exert a cumulative effect. In recent years, cumulative measurement of oxidative stress parameters using the TOS method is preferred rather than measuring oxidative stress parameters individually. Under physiological conditions, the organism is protected from free oxygen radicals by the antioxidant defense systems that metabolize these radicals. It is well known that oxygen radicals that are produced in ischemic tissues cannot be fully eliminated by the antioxidant defense system, and free radical reactions are among the major causes of tissue injury following ischemia [36]. While it is possible to measure the antioxidant parameters individually, a kit has been developed to determine the total antioxidant status levels. This provides information about enzymatic and nonenzymatic antioxidants.

Aytacoglu et al. have performed occlusion-reperfusion in infrarenal abdominal aorta of rats and demonstrated that TAS levels are higher in the iloprost-treated group compared to the nontreated group [14]. In a similar study, Koksel et al. demonstrated that iloprost leads to a significant increase in TAS levels [11]. Koca et al., on the other hand, performed arthroscopic knee surgery in patients and demonstrated higher TAS levels and lower TOS levels in the NAC-treated group [37].

Rabus et al. measured TAS and TOS levels in tissues and plasma in rheumatic and degenerative heart valve diseases and did not find a significant difference in tissue and plasma TOS levels between the groups. While the authors did not find a significant difference in plasma TOS levels, they demonstrated that the tissue TAS levels were significantly different [38]. Similarly, we only analyzed plasma TAS levels and did not find a significant difference between the iloprost-treated group and NAC-treated group. Considering the degree of reflection of tissue injury to the periphery and the possible differences between the tissues, it is clear that greater knowledge and research are required on different factors that affect TAS levels. Overall, biochemical findings showed that iloprost and NAC were effective in reducing acute ischemia-reperfusion injury, and iloprost was more effective compared to NAC.

## 5. Conclusion

In conclusion, iloprost and N-acetylcysteine protect muscular tissue against ischemia-reperfusion, and the administration of these agents decreases ischemia-related tissue injury. Nevertheless, other systematic effects and beneficial cellular effects of these drugs should be investigated in further clinical trials.

## Figures and Tables

**Table 1:** Variance analysis of biochemical findings. Total antioxidant status (TAS), Total Oxidant Status (TOS), creatine kinase (CK), and lactate dehydrogenase (LDH).

Parameters	Groups	Mean ± standard deviation	*P* value
TAS	Group 1	1.764 ± 0.09	**<0.05**
	Group 2	1.065 ± 0.10	
	Group 3	1.349 ± 0.05	
	Group 4	1.275 ± 0.049	

TOS	Group 1	10.57 ± 2.51	**<0.05**
	Group 2	56.73 ± 5.02	
	Group 3	27.52 ± 3.61	
	Group 4	39.92 ± 2.99	

CK	Group 1	5082 ± 1056	**<0.05**
	Group 2	28738 ± 2117	
	Group 3	16117 ± 1720	
	Group 4	20942 ± 1549	

LDH	Group 1	2231 ± 218	**<0.05**
	Group 2	6090 ± 406	
	Group 3	3937 ± 148	
	Group 4	4786 ± 313	

**Table 2:** Histological findings of all groups.

	Control group	I-R group	Iloprost group	NAC group	
Loss of striation	0	1	2	2	
Uncertainties in the peripheral nucleus	0	0	0	0	NS
Necrosis	0	2	1	1	
Cellular swelling	0	1	0	1	
Edema	0	2	1	2	
Fibrosis	0	2	1	2	
Leukocyte accumulation in capillaries	0	2	2	1	
Congestion	0	2	2	1	
Hemorrhage	0	0	0	0	NS
PMNL infiltration	0	3	1	1	
Monocyte-macrophage infiltration	0	2	1	1	

NS: not significant.

**Table 3:** Comparison of histopathological results.

Parameters	Groups	Median (25–75 perc.)	Statistical test (*H*)	*P* value
Loss of striation	Group 1	0 (0-0)	14.467	**<0.05**
	Group 2	1.5 (1-2)		
	Group 3	0 (0-1)		
	Group 4	1 (0-1)		

Faint peripheral nuclei	Group 1	0 (0-0)	3.932	NS
	Group 2	0 (0-0)		
	Group 3	0 (0-0)		
	Group 4	0 (0-0)		

Necrosis	Group 1	0 (0-0)	19.172	**<0.05**
	Group 2	1.5 (1-2)		
	Group 3	0.5 (0-1)		
	Group 4	1 (0-1)		

Cellular swelling	Group 1	0 (0-0)	11.766	**<0.05**
	Group 2	1 (0-1)		
	Group 3	0 (0-0)		
	Group 4	0 (0-1)		

Edema	Group 1	0.5 (0-1)	9.415	**<0.05**
	Group 2	2 (1–3)		
	Group 3	1 (0–2)		
	Group 4	1.5 (1-2)		

Fibrosis	Group 1	0 (0-0)	17.759	**<0.05**
	Group 2	2 (1-2)		
	Group 3	1 (0–2)		
	Group 4	2 (1–3)		

Leukocyte accumulation in capillaries	Group 1	0 (0-0)	13.064	**<0.05**
	Group 2	2 (2-2)		
	Group 3	0.5 (0–2)		
	Group 4	0 (0-1)		

Congestion	Group 1	0 (0-0)	15.957	**<0.05**
	Group 2	2 (1-2)		
	Group 3	2 (2-3)		
	Group 4	1.5 (0–2)		

Hemorrhage	Group 1	0 (0-0)	2.274	NS
	Group 2	0 (0-0)		
	Group 3	0 (0-0)		
	Group 4	0 (0-0)		

PMNL infiltration	Group 1	0 (0-0)	21.263	**<0.05**
	Group 2	3 (3-3)		
	Group 3	1.5 (1-2)		
	Group 4	0 (0–2)		

Monocyte-macrophage infiltration	Group 1	0 (0-1)	15.755	**<0.05**
	Group 2	2.5 (2-3)		
	Group 3	1 (0–2)		
	Group 4	0.5 (0–2)		

NS: not significant.

## Limitations of Study

There are two major limitations that need to be addressed regarding the present study. This study described experimental I/R model in healthy rats. Therefore, these results reflect acute results and these results should be confirmed in human subjects. The other limitation concerns the sample size of study. The sample size is small to give the high statistical power. However, ethical approval could be obtained to perform the study with a small number of animals.
